# Calcium/calmodulin-stimulated adenylyl cyclases 1 and 8 regulate reward-related brain activity and ethanol consumption

**DOI:** 10.1007/s11682-018-9856-6

**Published:** 2018-03-28

**Authors:** Kelly E. Bosse, Farhad Ghoddoussi, Ajay T. Eapen, Jennifer L. Charlton, Laura L. Susick, Kirt Desai, Bruce A. Berkowitz, Shane A. Perrine, Alana C. Conti

**Affiliations:** 1 Research & Development Service, John D. Dingell VA Medical Center, Detroit, MI USA; 2 Department of Neurosurgery, Wayne State University School of Medicine, Detroit, MI USA; 3 Department of Anesthesiology, Wayne State University School of Medicine, Detroit, MI USA; 4 Department of Psychiatry and Behavioral Neurosciences, Wayne State University School of Medicine, Detroit, MI USA; 5 Department of Anatomy and Cell Biology, Wayne State University School of Medicine, Detroit, MI USA; 6 Department of Ophthalmology, Wayne State University School of Medicine, Detroit, MI USA; 7 Department of Neurosurgery, Wayne State University, 4646 John R St., Detroit, MI 48201 USA

**Keywords:** Adenylyl cyclase, Calcium, Cortico-basal ganglia-thalamic, Ethanol, Magnetic resonance imaging, Manganese

## Abstract

Evidence suggests a predictive link between elevated basal activity within reward-related networks (e.g., cortico-basal ganglia-thalamic networks) and vulnerability for alcoholism. Both calcium channel function and cyclic adenosine monophosphate (cAMP)/protein kinase A-mediated signaling are critical modulators of reward neurocircuitry and reward-related behaviors. Calcium/calmodulin-stimulated adenylyl cyclases (AC) 1 and 8 are sensitive to activity-dependent increases in intracellular calcium and catalyze cAMP production. Therefore, we hypothesized AC1 and 8 regulate brain activity in reward regions of the cortico-basal ganglia-thalamic circuit and that this regulatory influence predicts voluntary ethanol drinking responses. This hypothesis was evaluated by manganese-enhanced magnetic resonance imaging and chronic, intermittent ethanol access procedures. Ethanol-naïve mice with genetic deletion of both AC1 and 8 (DKO mice) exhibited bilateral reductions in baseline activity within cortico-basal ganglia-thalamic regions associated with reward processing compared to wild-type controls (WT, C57BL/6 mice). Significant activity changes were not evident in regions either outside of the cortico-basal ganglia-thalamic network or within the network that are not associated with reward processing. Parallel studies demonstrated that reward network hypoactivity in DKO mice predicted a significant attenuation in consumption and preference levels to escalating ethanol concentrations (12, 20 and 30%) compared to WT mice, an effect that was maintained over extended access (14 sessions) to 20% ethanol. Summarizing, these data support a contribution of AC1 and 8 in cortico-basal ganglia-thalamic activity and the predictive value of this regulatory influence on ethanol drinking behavior, which merits the future evaluation of calcium-stimulated ACs in the neural processes that engender vulnerability to maladaptive alcohol drinking.

**Electronic supplementary material:**

The online version of this article (10.1007/s11682-018-9856-6) contains supplementary material, which is available to authorized users.

## Introduction

In recent years, studies identifying the neurocircuitry mediating alcohol action have strongly implicated the major nuclei and connectivity of the cortical-basal ganglia (BG)-thalamic network in alcohol pathophysiology. Concerted activity across this anatomically vast network was shown to have a specific role in mediating reward-guided action and its aberrant regulation in an alcohol-addictive state (Balleine et al. 2015; Noori et al. 2012; Yager et al. 2015). Thus, the transition from recreational to compulsive drug seeking, associated with addiction, likely involves dysregulation of integrative cortico-BG-thalamic circuits, leading to loss of control over goal-directed actions, dysfunction of habit learning processes and subsequent motivational and emotional changes associated with extended drug exposure (Balleine et al. 2015; Noori et al. 2012). While human imaging studies have extensively characterized functional alterations associated with alcohol abuse, there is a growing interest in defining if these alterations are the sole result of chronic alcohol use or represent innate factors which predict biological propensity for alcohol. MRI studies in individuals with familial risk for alcohol dependence showed atypical activation of specific cortico-BG-thalamic regions in response to alcohol cues (Kareken et al. 2010), as well as in a monetary incentive delay task (Andrews et al. 2011) or a gambling task (Acheson et al. 2009), linking activation of this circuit to abnormal reward sensitivity and elevated vulnerability for alcohol abuse. Correspondingly, altered resting brain function was also observed in focal cortico-limbic and thalamic areas in ethanol-naïve animals with genetic predisposition to alcohol, highlighting the predictive value of heritable neurofunctional alterations in alcohol susceptibility (Gozzi et al. 2013).

Several mechanisms have been implicated in the regulation of excitatory input function and responsiveness underlying reward-guided action selection in the cortico-BG-thalamic network, including calcium (Ca^2+^) channel activity. Multiple lines of evidence indicate a major contribution of Ca^2+^ flux through voltage-gated Ca^2+^ channels in the neuronal and behavioral responses to alcohol (Walter and Messing 1999). Specifically, L-type Ca^2+^ channels (LTCCs) are shown to mediate ethanol drinking behavior (De Beun et al. 1996; Fadda et al. 1992; Walter and Messing 1999) and to play a particularly essential role in behavioral stimulation induced by ethanol compared to other psychoactive drugs (Balino et al. 2010), though the intracellular mediators of the interaction between LTCCs and ethanol are presently unclear. The cyclic adenosine monophosphate (cAMP)/protein kinase A (PKA) signaling pathway is an important cellular substrate in the neurobiological response to ethanol (Ron and Messing 2013) that was recently shown to be regulated by Ca^2+^-dependent mechanisms (Balino et al. 2014). Specifically, evidence suggests Ca^2+^/calmodulin-stimulated adenylyl cyclase (AC) isoforms 1 and 8 mediate the activation of PKA-dependent signals by ethanol (Conti et al. 2009a; Maas et al. 2005b) and mice lacking both isoforms (i.e. double-knockout (DKO) mice) display aberrant stimulatory responses to ethanol (Conti et al. 2012). Human studies have further identified abnormalities in AC enzymes in alcohol abusers and those biologically vulnerable to this disorder (Hoffman et al. 2002), and selective increases in the neuronal expression of AC1 and 8 in reward-related brain regions of human alcoholics (Yamamoto et al. 2001). Together, these studies suggest intrinsic Ca^2+^-stimulated AC/cAMP/PKA activity represents a critical determinant for individual responsiveness to chronic ethanol and, moreover, a trait marker for predisposition to alcoholism, perhaps through influencing the permissive state of brain reward systems to activation and Ca^2+^-dependent synaptic modifications.

The most widely available imaging modality for investigating the function of the brain reward system is blood oxygenation level-dependent (BOLD) functional magnetic resonance imaging (fMRI) (Schulte et al. 2012). While highly valuable, fMRI’s major limitations include low spatial resolution and a readout that can only infer neuronal activity based on hemodynamic indices. A more recent advancement to this imaging approach is manganese (Mn^2+^)-enhanced MRI (MEMRI), which addresses these concerns by mapping the accumulation of the contrast agent, Mn^2+^ ions, from awake and freely moving animals with high spatial resolution. Mn^2+^ is known to enter activated neurons through Ca^2+^ channels, primarily the LTCCs (Lin and Koretsky 1997; Silva and Bock 2008). As the cellular efflux of Mn^2+^ is much slower than in its influx, MEMRI allows for the collection of very high resolution activation maps in vivo, permitting quantitative estimates of global function not readily attainable with post-mortem proxies of brain activity, such as immediate early gene induction (Hattori et al. 2013). MEMRI has also been extensively employed for functional analysis of resting-state brain activity to study gene and trait-dependent neural phenotypes in transgenic and selective breeding lines (Lutkenhoff et al. 2012; McGuire et al. 2013; Perez et al. 2013; Thinschmidt et al. 2016).

Here, we used a two-arm study design to test the hypothesis that AC1 and 8 regulate basal brain activity in regions of the cortico-BG-thalamic circuit related to establishment of alcohol reinforcement and that this regulatory influence predicts voluntary ethanol drinking responses. In the first arm, we evaluated baseline neural activity within regions of the cortico-BG-thalamic network that are implicated in regulating alcohol action (Balleine et al. 2015; Noori et al. 2012; Yager et al. 2015) in mice lacking Ca^2+^-stimulated AC1 and 8 and wild-type (WT) controls using MEMRI as a measure of Ca^2+^ activity. In the second arm, we evaluated genotypic differences in ethanol drinking behavior following access to increasing ethanol concentrations (3, 6, 12, 20, 30% *v*/*v*) and over extended access to a single ethanol concentration (20% *v*/*v*) using a chronic, intermittent two-bottle choice procedure (Hwa et al. 2011; Melendez 2011).

## Materials and methods

### Animals

The generation of DKO mice using a targeted mutagenesis strategy has been described previously (Wong et al. 1999; Wong and Storm 2002; Wu et al. 1995). DKO mice were backcrossed more than 10 generations onto a C57BL/6 background (C57BL/6 mice obtained from The Jackson Laboratory), with homogeneity on the C57BL/6 background established by comparison of polymorphic markers between C57BL/6 and 129 mouse strains. Disruption of the AC1 and AC8 genes were directly confirmed through assessing Ca^2+^-stimulated AC activity in several brain regions, including the neocortex and striatum (DiRocco et al. 2009; Wu et al. 1995). Age-matched progeny of DKO homozygous mutants and WT (C57BL/6) mice (male, 12–13 weeks old) were bred and raised in-house. Subjects for experimental cohorts were randomly assigned from several different litters. Mice were maintained in standard microisolator cages under controlled conditions (~ 24 °C; lights on 06:00–18:00 h; 35–40% humidity) with constant free access to food and water. All procedures were approved by the Wayne State University Institutional Animal Care and Use Committees. Animal care and use followed NIH Office of Laboratory Animal Welfare guidelines and was overseen by AAALAC accredited facilities at Wayne State University.

### MEMRI procedure

Three separate cohorts of mice were injected intraperitoneally (i.p.) with saline (no-Mn^2+^ controls) or manganese (66 mg MnCl_2_ ∙4H_2_O/kg, dissolved in phosphate-buffered saline to a concentration of 0.1 M) and imaged 24 h later. Systemic administration of similar MnCl_2_ doses was shown to produce robust signal enhancement with optimal regional contrast ~ 24 h after injection with no detectable side effects (Bissig and Berkowitz 2009, 2011; Lee et al. 2005; Silva et al. 2004). Prior to scanning, mice were anesthetized with urethane (2.2 ± 0.2 mL/kg, i.p., 36% solution in saline) to achieve long-lasting anesthesia without major effects on physiologic parameters. Anesthetized mice were secured using blunted ear-bars and a tooth-bar. Body temperature was maintained using a heated recirculating water blanket. Scans were performed on a 7-T Bruker (Billerica, MA) ClinScan system with a Siemens (Munich, Germany) console using a transmit-only whole-body coil and a receive-only surface coil (four-element phased array for mouse brain), which was placed dorsal to the head (Bissig and Berkowitz 2009, 2011; Perrine et al. 2015). Images were acquired using a turbo-fast low-angle (FLASH) sequence, in which scans were obtained with and without a slice-selective inversion pulse generated magnetization prepared rapid acquisition gradient echo (MPRAGE) and proton density-weighted (PDGE) images with principally mutual parameters (flip angle, 3°; TE = 3.03 ms, NA = 1; echo spacing, 7.77 ms; matrix size, 192 × 192 × 112; field of view, 2.50 × 2.50 × 2.91 cm^3^; resolution, 130 × 130 × 260 µm). MPRAGE imaging was completed in 8 min and PDGE imaging was completed in 3 min, 12 s. For each mouse, the sequences of MPRAGE and PDGE image acquisition were performed twice and interleaved. Complete sequences were acquired for every animal in a counter-balanced order according to genotype. Immediately following image sequence acquisition, animals were euthanized by decapitation, while remaining under anesthesia.

### Image analysis

Prior to image analysis, PDGE and MPRAGE images were registered and a ratio image obtained by dividing the signal intensity from MPRAGE images with the corresponding PDGE images on a voxel-by-voxel basis (Bissig and Berkowitz 2009, 2011; Perrine et al. 2015). The resulting MPRAGE/PDGE ratio is weighted heavily by tissue *T*_1_ and is largely devoid of other variables that contribute to intensity field bias, including tissue proton density, receive coil distance and *B*_1_ inhomogeneity (Van de Moortele et al. 2009). Whole-brain 3D sets of images from each subject (MPRAGE, PDGE and ratio) were uploaded in ImageJ (Rasband, W.S., ImageJ, U. S. National Institutes of Health, Bethesda, Maryland, USA, http://rsb.info.nih.gov/ij/, 1997–2008). Identification of neuroanatomical landmarks and construction of 2D regions of interest (ROIs) templates were performed with careful comparison of MR images with the mouse brain atlas (Paxinos and Franklin 2001). ROI signal intensities were similarly determined for a separate group of no-Mn^2+^ controls (injected with saline only) to verify the presence of Mn^2+^ enhancement. ROIs were selected from the cortico-BG-thalamic circuit based on their proposed role in ethanol reward (Balleine et al. 2015; Dudek et al. 2015; Noori et al. 2012; Vilpoux et al. 2009). Prominent landmarks included white matter tracts (e.g. corpus callosum, anterior and posterior commissures and fasciculus retroflexus), the overall shape of the brain and ventricle location, and defined gray matter structures (e.g. caudate putamen). The atlas-based, user-defined ROI templates were used to maintain uniform quantification of signal intensities from *T*_1_-weighted ratio images across subjects. Muscle normalization is an established method to account for variables that could influence signal intensity but are independent of neuronal activity, such as receiver coil signal intensity gradients or inter-individual variances in systemic Mn^2+^ processing (e.g. liver sequestration). Thus, average signal intensities for each ROI were normalized to the mean signal intensity recorded for the temporalis muscle tissue (located adjacent to the skull, averaged from 10 bilateral regions) within subjects for both the Mn^2+^ and no-Mn^2+^ condition prior to group comparisons. Prior to muscle normalization, signal intensities from lateral ROIs were averaged from the left and right hemisphere for each subject.

### Intermittent two-bottle choice procedure

Drinking studies were evaluated in two separate cohorts of mice, one to measure drinking with extended access to 20% (*v*/*v*) ethanol and the other to measure ethanol concentration-dependent drinking. Both cohorts were acclimated to single-housing conditions for 1 week prior to initiating the drinking procedure. Each study followed a standard intermittent two-bottle choice procedure for ethanol access in which mice were given 24-h concurrent access to one bottle containing ethanol and one bottle containing tap water, followed in the next 24-h session with access to two bottles containing tap water (Hwa et al. 2011). Ethanol and water sessions were conducted on Monday (M), Wednesday (W), and Friday (F) with 24-h (Tuesday and Thursday) or 48-h (weekend) ethanol-deprivation periods (access to two water bottles) between drinking sessions. Drinking sessions began 2 h before the dark cycle (bottles in at 16:00 h) and bottle placements were alternated between each session to control for side preference. Bottle weights were recorded daily and levels of ethanol and water consumption, as well as preference for ethanol to total fluid intake, were quantified. Body weight was measured prior to the first session and monitored weekly during the study. Ethanol intake levels (g/kg) were calculated with the corresponding week’s weight. To measure extended access, only one concentration of ethanol, (20% *v*/*v*, in tap water) was supplied for the duration of the study (14 access sessions), while the concentration of ethanol was increased each week (3 access sessions; M, W and F) from 3, 6, 12, 20 and 30% (*v*/*v*, in tap water) for the concentration-dependent study.

### Statistical analysis

Data and statistical analyses were performed using Excel and GraphPad Prism 6 software. All values are reported as mean ± SEM and the criterion for statistical significance was p ≤ 0.05 (α = 0.05). Independent Student’s two-tailed t-tests with Holm-Sidak correction were used to evaluate both normalized MEMRI signal intensities for individual ROIs and average/sum 20% ethanol consumption and preference values. Two-way ANOVA with Sidak’s multiple comparison analysis was used to compare combined muscle signal intensities across WT and DKO mice in no-Mn^2+^ and Mn^2+^-treated cohorts. Repeated measure (RM) ANOVAs were used to compare genotype (between subjects factor) and time or ethanol concentration (within subjects factor) effects in the two-bottle choice intake studies. Group sizes for experiments were calculated using a priori power analysis (power (1-β) = 0.8, α = 0.05) based on estimates of effect size and variance obtained from preliminary data and published literature (Dudek et al. 2015; Perrine et al. 2015).

#### Data availability

The datasets generated and/or analyzed during the current study are available from the corresponding author on reasonable request.

## Results

In all regions analyzed, normalized signal intensity was significantly increased in Mn^2+^-treated cohorts (WT and DKO, n = 13/genotype) compared to no-Mn^2+^ (saline-treated) controls (WT, n = 7; DKO, n = 6; see Online Resource 1) with respect to genotype (p < 0.05), indicating that detectable levels of Mn^2+^ accumulation were achieved with the treatment protocol. ANOVA comparison of mean signal intensities for combined muscle regions revealed a significant main effect of treatment (F(1,35) = 74.08, p < 0.0001), but no effect of genotype (F(1,35) = 0.987, p = 0.33) or interaction (F(1,35) = 0.906, p = 0.34), denoting that similar signal enhancement within normalization regions were observed following Mn^2+^ administration in WT (WT Mn^2+^ vs. WT no- Mn^2+^, p < 0.05) and DKO mice (DKO Mn^2+^ vs. DKO no- Mn^2+^, p < 0.05) (data not shown).

### DKO mice have reduced basal MEMRI activity in reward-related regions

**Fig. 1:**
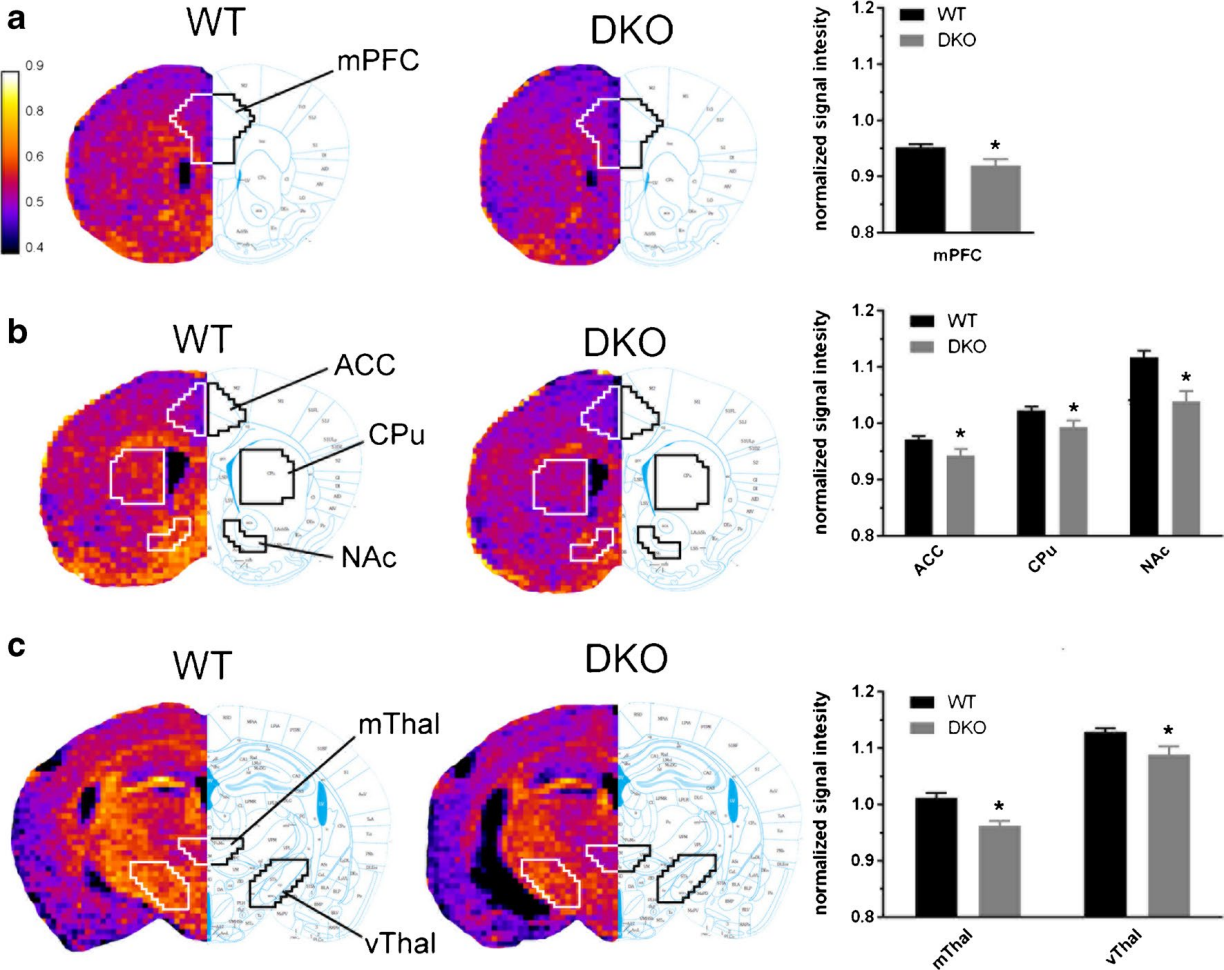
Baseline neurofunctional activity in multiple reward-related brain regions is significantly decreased in DKO mice, compared to WT mice (n = 13/genotype). Manganese (Mn^2+^)-enhanced MRI (MEMRI) was conducted following 24 h of Mn^2+^ uptake and analyzed from magnetization prepared rapid acquisition gradient echo/proton density weighted (MPRAGE/PDGE) images of coronal sections containing the **a**, medial prefrontal cortex (mPFC); **b**, anterior cingulate cortex (ACC), anterior caudate putamen (CPu), and nucleus accumbens (NAc); **c**, medial thalamus (mThal) and ventral lateral thalamus (vThal). Each panel shows: (1) ROI placements on a representative sample ratio (MPRAGE/PDGE) image, utilizing a pseudocolor to indicate signal intensity and Mn^2+^ uptake, alongside the corresponding mouse brain atlas image, and (2) average normalized signal intensities (mean ± SEM) for each ROI. *p ≤ 0.05, compared to WT controls

To test the first arm of our hypothesis that AC1 and 8 regulate basal brain activity in regions involved in alcohol reinforcement, anatomical ROI analyses were focused on regions of the cortico-BG-thalamic circuit suggested to facilitate ethanol reward responding (Balleine et al. 2015; Dudek et al. 2015; Noori et al. 2012; Vilpoux et al. 2009). Fig. 1a–c show: (1) ROI outlines on representative pseudocolor ratio images from a Mn^2+^-treated WT and DKO mouse (left hemisphere) and the corresponding mouse atlas image (right hemisphere) and (2) average normalized MEMRI signal intensities, progressing anterior to posterior (n = 13/genotype). Relative to WT mice, normalized signal intensities measured 24 h following Mn^2+^ administration were significantly decreased in DKO mice within the medial prefrontal cortex (mPFC; t_13_ = 3.13, p = 0.002), anterior cingulate cortex (t_13_ = 2.86, p = 0.005), anterior caudate putamen (t_13_ = 2.53, p = 0.012), nucleus accumbens (NAc, t_13_ = 5.35, p < 0.001), medial thalamus (t_13_ = 3.40, p < 0.001) and ventral lateral thalamus (t_13_ = 2.72, p = 0.007). In contrast, no differences in MEMRI signal intensity were detected among WT and DKO mice in either the anterior (signal intensity in WT: 9.499 ± 0.007, DKO: 9.301 ± 0.010, p = 0.142) or posterior (WT: 9.566 ± 0.007, DKO: 9.324 ± 0.011, p = 0.059) secondary motor cortex that border the cingulate cortex, or the habenula (WT: 1.216 ± 0.013, DKO: 1.169 ± 0.022, p = 0.078) located in the posterior-medial aspect of the dorsal thalamus.

### Decreased voluntary ethanol consumption in DKO mice

**Fig. 2:**
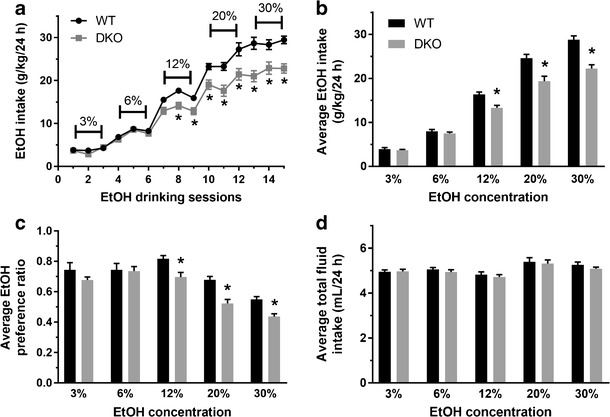
Voluntary ethanol (EtOH) intake (**a,b**), EtOH preference ratio (**c**) and total fluid (EtOH + water) intake (**d**) in WT and DKO mice (n = 8–10/genotype) following access to increasing ethanol (EtOH) concentrations (3, 6, 12, 20 and 30%, v/v in tap water, 3 sessions each) assessed in an intermittent, two-bottle choice procedure. Concentration-dependent reductions in EtOH consumption and preference were observed in DKO mice during access to the highest EtOH solutions tested, with no change in total fluid intake (mean ± SEM), compared to WT mice. *p ≤ 0.05, compared to WT controls

Next, the predictive nature of baseline hypoactivity within reward-associated regions of the cortico-BG-thalamic network in DKO mice on voluntary ethanol drinking was evaluated. Separate studies were performed to measure ethanol intake and preference over a range of ethanol concentrations (3, 6, 12, 20 and 30% *v*/*v*, 3 sessions each) and over an extended access period (20% *v*/*v*, 14 access sessions) using an intermittent, two-bottle choice procedure. Ethanol consumption (Fig. 2a, b), preference (Fig. 2c) and total fluid intake (Fig. 2d) were assessed in WT and DKO mice following free-choice access to increasing ethanol concentrations (n = 8–10/genotype). Two-way RM ANOVA analysis revealed significant main effects of genotype and time or concentration, as well as significant interactions for both for consumption (genotype: F(1,16) = 23.29, p = 0.0002; time: F(14, 224) = 241.2, p < 0.0001; concentration F(4,64) = 500.4, p < 0.0001; genotype x time: F(14,224) = 5.89, p < 0.0001; genotype x concentration: F(4,64) = 11.75, p < 0.0001) and preference (genotype: F(1,16) = 12.1, p = 0.015; time: F(14, 224) = 25.3, p < 0.0001; concentration F(4,64) = 39.91, p < 0.0001; genotype x time: F(14,224) = 3.07, p < 0.0002; genotype x concentration: F(4,64) = 2.60, p = 0.044). WT mice displayed a proportional escalation in ethanol intake with increasing concentrations of ethanol, with a decrease in preference ratio observed at only the highest ethanol concentration tested (30%, p < 0.05). Post-hoc analysis determined that DKO mice had a parallel reduction in consumption and preference levels compared to WT mice selectively at high ethanol concentrations (12, 20 and 30%, p < 0.05 for each). Indeed, DKO mice demonstrated little to no preference for the two highest ethanol concentrations tested compared to a decreased, but sustained, preference response in WT mice (20%, WT: 0.68 ± 0.02, DKO: 0.52 ± 0.03; 30%, WT: 0.55 ± 0.02, DKO: 0.43 ± 0.02). While total fluid (ethanol + water) intake varied slightly with ethanol concentration (main effect: F(4,64) = 13.68, p < 0.01), no effect of genotype (F(1,16) = 0.47, p = 0.50) or interaction (F(4,64) = 0.36, p = 0.84) was observed, indicating that the attenuation of drinking in DKO mice was not the result of overall reductions in fluid consumption.

**Fig. 3:**
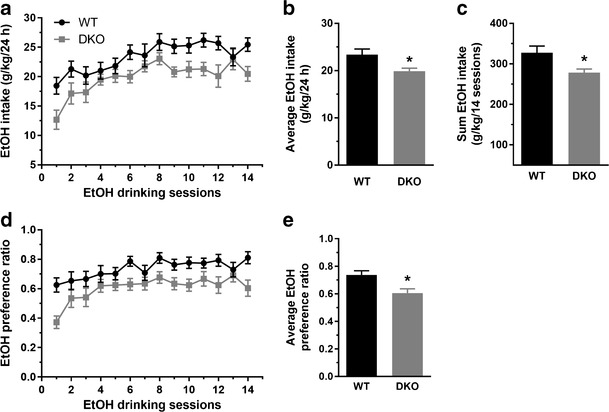
Voluntary ethanol (EtOH) intake (**a,b,c**) and EtOH preference ratio (**d,e**) in WT and DKO mice (n = 8/genotype) across 14 EtOH access sessions assessed in an intermittent, two-bottle choice procedure. Intermittent access to 20% EtOH (*v*/*v* in tap water) induced a gradual increase in EtOH intake and preference (mean ± SEM). DKO mice displayed a significantly attenuated average EtOH consumption and preference together with a reduced total EtOH intake for the duration of the study (sum) relative to WT mice. *p ≤ 0.05, compared to WT controls

To establish if the genotypic reduction in ethanol drinking responses was maintained over extended access, consumption (Fig. 3a, b, c) and preference ratios (Fig. 3d, e) were assessed in a separate cohort of WT and DKO mice given intermittent access to 20% ethanol for 14 sessions (n = 8/genotype). Intermittent access to this ethanol concentration was shown to induce a gradual escalation in intake that reaches a stable baseline in rodents without the need for an initiation method (Hwa et al. 2011; Melendez 2011). Two-way RM ANOVA analysis revealed significant main effects of genotype and time, but no interaction, for both ethanol intake (genotype: F(1,13) = 6.95, p = 0.020; time: F(13, 182) = 11.10, p < 0.0001; no interaction: F(13,182) = 1.29, p = 0.22) and preference (genotype: F(1,13) = 7.931, p = 0.015; time: F(13, 182) = 7.931, p < 0.0001; no interaction: F(13,182) = 1.303, p = 0.215). Independent t-test analysis of ethanol consumption and preference during the initial session of voluntary access revealed a marked reduction in both parameters in DKO mice compared to WT mice (each p < 0.05). Average ethanol consumption (23.4 ± 1.2 g/kg/24 h) and preference ratio (0.74 ± 0.03) in WT mice over the course of the study (14 sessions) were consistent with the range of drinking levels reported in male C57BL/6J mice (Hwa et al. 2011; Melendez 2011). Comparatively, DKO mice displayed a significant attenuation of both average ethanol intake (19.9 ± 0.6 g/kg/24 h, p < 0.05) and preference ratio (0.61 ± 0.03, p < 0.01) relative to WT mice. This reduction in average ethanol intake in DKO mice was mirrored by a significant decrease in the sum of ethanol consumption over the duration of the 14 session study (WT mice: 327 ± 16 g/kg; DKO mice: 278 ± 8 g/kg; p < 0.05) and compensated by an increase in water consumption (RM ANOVA: significant main effect of genotype: F(1,13) = 26.72, p < 0.001 and time: F(13,182) = 3.904, p < 0.001; no interaction: F(13,182) = 0.604, p = 0.849), indicative of an active avoidance response to ethanol rather than a non-specific inhibition of consummatory behavior. Average total fluid intake did not significantly differ between WT (5.52 ± 0.05 mL/24 h) and DKO mice (5.38 ± 0.05 mL/24 h) as determined by RM ANOVA (significant main effect of time: F(13,182) = 2.709, p > 0.01; but no effect of genotype: F(1,13) = 1.300, p = 0.273 or interaction: F(13,182) = 1.436, p = 0.146).

## Discussion

In this study, we find evidence to support both arms of our working hypothesis that AC1 and 8 regulate basal brain activity in reward-related cortico-BG-thalamic regions and that hypoactivation of these regions in mice lacking AC1 and 8 predicts overall reductions in alcohol intake, which are particularly evident at high ethanol concentrations. While future studies are necessary to establish a direct relationship between these outcomes, our findings are consistent with cortico-BG-thalamic involvement in alcohol behavioral responding (Balleine et al. 2015; Dudek et al. 2015; Noori et al. 2012; Vilpoux et al. 2009; Yager et al. 2015) and previous evidence for aberrant resting-state function in animal models of genetic predisposition to alcohol dependence (Gozzi et al. 2013). Further, our findings extend behavioral phenotypes, including increased sedation and reduced locomotor activation (Conti et al. 2012; Maas et al. 2005b), and neural outcomes related to PKA activation, neuronal reactivation and neuroapoptotic pathways (Conti et al. 2009b; Maas et al. 2005a; Moulder et al. 2008) identified with ethanol exposure in DKO mice, supporting a biological role for calcium-stimulated ACs in the pathological response to ethanol.

The present study utilized MEMRI to map areas of differential activity levels within the cortico-BG-thalamic circuit under baseline, resting-state conditions in WT and DKO mice. Our results in DKO mice demonstrate significant reductions in Mn^2+^ signal intensity within regions of this circuit that are integral for reward, associated with heritable brain abnormalities related to alcohol predisposition (Gozzi et al. 2013), and exhibit Ca^2+^/calmodulin-stimulated AC activity and AC1 and 8 expression (Cali et al. 1994; Conti et al. 2007; DiRocco et al. 2009; Xia et al. 1991). The most pronounced effects were observed in the medial thalamus and NAc, the latter of which has long been recognized as critical for establishment of acute reinforcing effects of ethanol (Koob et al. 1998; McBride et al. 1999). One consideration is that the hypoactivity observed in DKO mice could result from widespread changes in Ca^2+^ entry. The observation that significant reductions in neural activity in DKO mice were not generalizable across the entire brain contradicts this supposition. For instance, no differences between WT and DKO mice were detected in the habenula, which is associated with behavioral responding to ethanol (Zuo et al. 2015) and a region in which AC8 is strongly expressed (Conti et al. 2007), but is outside of the cortical-BG-thalamic network. Likewise, activity in regions of the motor cortex that lie within the cortical-BG-thalamic network, but do not play a vital role in reward responding were unaffected. Predominately, the neuronal effects resulting from loss of Ca^2+^-stimulated AC activity have been characterized in the hippocampus. These studies reveal limited evidence for the contribution of AC1 and 8 to basal Ca^2+^-dependent synaptic function, but, rather their regulatory role in electrophysiologic properties under evoked, activity-dependent conditions, such as pre- and post-synaptic long-term potentiation (Conti et al. 2009a; Moulder et al. 2008; Villacres et al. 1998; Wong et al. 1999). While our present data would not permit us to theorize which Ca^2+^-related neuronal functions would be impaired in DKO mice, the region-specific effects we observed with MEMRI support the concept that Ca^2+^-stimulated ACs maintain cellular function within reward circuits of the cortico-BG-thalamic network that has not previously been identified.

The intermittent, two-bottle choice assay used to evaluate alcohol drinking behavior induces a gradual escalation to excessive consumption levels (Hwa et al. 2011; Melendez 2011), which is thought to recapitulate many key features of clinical alcohol abuse (Carnicella et al. 2014). Using this procedure, we demonstrate that DKO mice had a detectable reduction in voluntary drinking responses during access to high ethanol concentrations (12, 20 and 30%). Additionally, reduced intake in DKO mice was observed over an extended access period to 20% ethanol, reflected by a decrease in both the average and sum of ethanol consumption and average preference ratio over 14 access sessions, together with a prominent attenuation on the initial day of ethanol exposure. As locomotor activity following acute ethanol was similarly blunted in DKO mice (Conti et al. 2012), these observations suggest AC1 and 8 regulate the magnitude of initial stimulatory and motivational responding to ethanol. Further, the degree of reduction in ethanol consummatory behavior in DKO mice is in line with comparable studies in transgenic models manipulating recognized components of reward circuits (Boehm et al. 2004; Cozzoli et al. 2012; El-Ghundi et al. 1998; Hall et al. 2001). The general finding that global genetic manipulations yield modest, albeit significant, decreases in voluntary ethanol consumption may reflect the contribution of compensatory pathways as well as support a gene network model so that no one component is sufficient to completely abate drinking. However, these considerations do not preclude the interpretation that these proteins are important in the reward associated processes evoked by ethanol, which is supported in the case of AC1 and 8 through the present finding that they regulate Ca^2+^-dependent neuroactivity in reward-related regions encoded by MEMRI. Moreover, our study expands upon previous evidence for concentration-dependent reductions in consumption behavior in DKO mice during continuous two-bottle choice access (Maas et al. 2005b), by utilizing the intermittent access model as there is some indication that intake under continuous access may not be entirely driven by the pharmacological effects of ethanol (Dole and Gentry 1984). These procedures are also thought to model different consumption patterns and motivational drive, consistent with evidence that they induce different genomic effects in the PFC (Osterndorff-Kahanek et al. 2013). Ethanol metabolism and taste preference for sucrose and quinine were unaltered in DKO mice (Maas et al. 2005b), indicating these parameters do not account for changes in drinking behavior. As the intermittent access procedure is thought to model the behavioral transition and neuroadaptive changes involved in the progression from social-like to excessive intake (Carnicella et al. 2014), our finding indicates a regulatory role of Ca^+ 2^-stimulated ACs in the adaptive responses to chronic ethanol associated with addiction.

Intriguingly, regions of attenuated function within the cortico-BG-thalamic circuit of DKO mice identified with MEMRI overlap with areas of activation targeted by ethanol. For example, alterations in neural activity in response to ethanol have been demonstrated in these regions by expressional changes in immediate early genes (Bachtell et al. 1999; Lin and Koretsky 1997; Vilpoux et al. 2009), and functional MEMRI (Dudek et al. 2015). Within the cortico-BG-thalamic network, mPFC and thalamus represent the major glutamatergic inputs into striatal areas, such as the caudate putamen and NAc, which, in turn, influence thalamic excitation and cortical feedback loops. The functional integration of these regions is increasing implicated in controlling aspects of goal-directed action planning, habit learning and consequent motivational and emotional dysregulation related to addiction (Balleine et al. 2015; Noori et al. 2012; Yager et al. 2015). Moreover, individuals at high-risk for developing alcohol dependence display heightened sensitivity in several cortico-BG-thalamic regions (Acheson et al. 2009; Andrews et al. 2011; Kareken et al. 2010) that were shown in the present study to have attenuated basal activation in ethanol-naïve DKO mice. Furthermore, the pronounced decrease in intake during initial exposure to ethanol in DKO mice is congruent with the concept that the degree of reward network reactivity prior to drug exposure may predict drug reward sensitivity (Acheson et al. 2009; Andrews et al. 2011; Kareken et al. 2010). While the idea that AC1 and 8 are recruited as part of the neuroadaptations that modulate behavioral responses to ethanol is conceptually supported by the present reduction in ethanol drinking behavior in DKO mice and previous evidence for their upregulation in the striatum of human alcoholic brains (Yamamoto et al. 2001), future MEMRI studies are warranted to longitudinally assess the trajectory and influence of Ca^2+^-stimulated ACs on alcohol-induced brain activation using the groundwork laid previously for this analysis (Dudek et al. 2015).

While other Ca^+ 2^/calmodulin sensors are known to regulate LTCC inactivation (Dittmer et al. 2014; Oliveria et al. 2012), this study is the first to our knowledge to identify the contribution of AC1 and 8 in Ca^+ 2^ channel maintenance in an intact system. Mn^2+^ has been shown to act as an LTCC surrogate for the divalent Ca^2+^ ion, thereby entering excitable cells primarily through voltage-gated LTCCs (Lin and Koretsky 1997; Silva and Bock 2008). In this way, Mn^2+^ accumulation reflects Ca^+ 2^-dependent neuronal activity mediated by LTCCs. Further, the present observations that DKO mice have reduced MEMRI signals and ethanol drinking levels highlight voltage-gated LTCCs as a conceivable mechanistic target coupling AC1 and 8 to ethanol action. LTCCs are integral in the cellular response to ethanol. An upregulation of LTCCs has been documented in neurons after sustained ethanol exposure (Katsura et al. 2006; Walter and Messing 1999). Further, pharmacologic studies show inhibitors of LTCCs reduce voluntary ethanol consumption (De Beun et al. 1996; Fadda et al. 1992; Walter and Messing 1999), which agrees with our concurrent findings in DKO mice of attenuated MEMRI signals, reflecting impaired LTCC function, in reward-associated regions and reductions in ethanol drinking. PKA phosphorylation has been shown to increase the open time and Ca^2+^ permeability of LTCCs (Kavalali et al. 1997; Murphy et al. 2014), providing a potential mechanism through which cAMP/PKA-mediated signaling induced by AC1 and 8 could influence LTCC activity. Further, to engender its neurobehavioral effects, ethanol induces PKA activation (Conti et al. 2009a; Maas et al. 2005b; Ron and Messing 2013) in a Ca^+ 2^-dependent manner (Balino et al. 2014). Combined with our present findings, this evidence suggests Ca^+ 2^-stimulated ACs link promotion of LTCC function in response to ethanol with Ca^+ 2^-dependent activation of PKA-stimulated downstream signaling, such that loss of AC1/8 would result in decreased ethanol responding through decoupling this interaction. More work is needed to test this mechanism in future studies, which could reconcile the observations indicating the key involvement of these AC isoforms in ethanol-evoked behaviors and signaling despite their inability to be directly activated by ethanol (Conti et al. 2009a; Maas et al. 2005b).

This study must be viewed in the context of a few considerations. A universal consideration in MEMRI studies centers around the potential toxicity of Mn^2+^ administration. Previous MEMRI studies have utilized Mn^2+^ doses as high as 175 mg/kg, with minimal side effects (Lee et al. 2005). Based on prior reports, the present dose (66 mg/kg) was considered optimal, providing excellent regional contrast in the absence of evident neuronal toxicity (Berkowitz et al. 2016; Bissig and Berkowitz 2009, 2011). The present study also utilized established methods, such as ratio (MPRAGE/PDGE) imaging and muscle normalization, to minimize potential confounds related to MRI (e.g. *B*_1_/*B*_0_ inhomogeneities and surface coil sensitivities) and Mn^2+^ administration (e.g. injection variability). We interpret the regional reduction in MEMRI signals in DKO mice as evidence for lower neural activity in areas involved in reward processing, but we recognize that Mn^2+^ can also be sequestered in glial cells (Sloot and Gramsbergen 1994). In support of our interpretation, expression of AC1 is known to be neuron specific (Xia et al. 1993), however, the cellular specificity of AC8 is less definitive. Biological factors, such as oxidative stress, tissue edema, cell density and neurodegeneration (Berkowitz et al. 2016; Silva et al. 2004), can affect the correlation between MEMRI signals and neural activity, potentially confounding our interpretation of group differences. At present, no evidence supports abnormal regulation of these parameters in adult DKO mice. Further, our finding that measurable Mn^2+^ accumulation was achieved in all brain regions irrespective of genotype (Mn^2+^ vs no-Mn^2+^ group), indicates non-specific, biological factors as an unlikely confound of the observed genotypic differences in uptake.

Collectively, we present novel evidence that AC1 and 8 contribute to Ca^2+^-mediated basal activity within reward processing pathways as well as consumption and preference responding to both escalating and stable concentrations of ethanol. Gradual escalation of more concentrated ethanol solutions mimics the evolution of human patterns of alcohol drinking preferences (Duncan et al. 2012) that are indicative of impaired control over drinking, a condition for which long-term and widely-efficacious clinical treatment options remain limited. Thus, future work will incorporate longitudinal assessment of MEMRI activity with intermittent two-bottle choice exposure to increasing ethanol concentrations and pharmacologic manipulations with the goal of understanding the link between Ca^2+^-stimulated ACs and LTCCs that may moderate this progression toward dysregulated alcohol intake to identify potential novel intervention strategies.

## Electronic supplementary material

Below is the link to the electronic supplementary material.

Supplementary material 1 (PDF 208 KB)
