# Supplementary material for: Calcium/calmodulin-stimulated adenylyl cyclases 1 and 8 regulate reward-related brain activity and ethanol consumption
Source: Brain Imaging Behav. 2018 Mar 28;13(2):396–407. doi: 10.1007/s11682-018-9856-6 (PMC6202255; doi:10.1007/s11682-018-9856-6)
Supplement: Supplementary file 1 — Supplementary material 1 (PDF 208 KB) [file 11682_2018_9856_MOESM1_ESM.pdf]

Calcium/calmodulin-stimulated adenylyl cyclases 1 and 8 regulate reward-related brain activity and ethanol consumption. Brain Imaging and Behavior. KE Bosse, et al. Corresponding author: AC Conti, John D. Dingell VAMC, Wayne State University, alana.conti@wayne.edu.

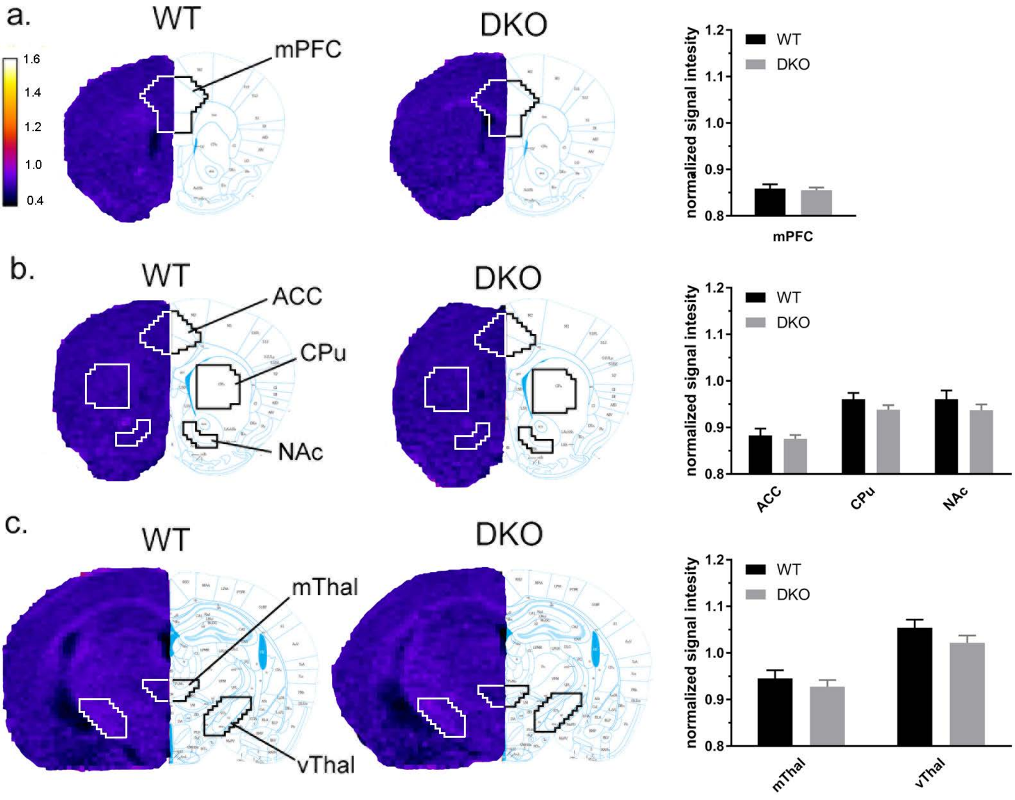

Calcium/calmodulin-stimulated adenylyl cyclases 1 and 8 regulate reward-related brain activity and ethanol consumption. Brain Imaging and Behavior. KE Bosse, F Ghoddoussi, AT Eapen, JL Charlton, LL Susick, K Desai, BA Berkowitz, SA Perrine and AC Conti

Corresponding author: AC Conti, John D. Dingell VAMC, Wayne State University, alana.conti@wayne.edu.

ESM. 1 Neurofunctional activity in no-manganese controls was comparable in saline-treated WT and DKO mice (n = 6-7/genotype). Scans were conducted 24 h after a single saline injection and analyzed from magnetization prepared rapid acquisition gradient echo/proton density weighted (MPRAGE/PDGE) images of coronal sections containing the **a,** medial prefrontal cortex (mPFC); **b,** anterior cingulate cortex (ACC), anterior caudate putamen (CPu), and nucleus accumbens (NAc); **c,** medial thalamus (mThal) and ventral lateral thalamus (vThal). Each panel shows: 1) ROI placements on a representative sample ratio (MPRAGE/PDGE) image, utilizing a pseudocolor to indicate signal intensity, alongside the corresponding mouse brain atlas image, and 2) average normalized signal intensities (mean ± SEM) for each ROI.
